# Complete Superior Orbicularis Oculi Blockade With Botulinum Toxin for Eyebrow Repositioning: A Pilot Study

**DOI:** 10.1111/jocd.71185

**Published:** 2026-09-16

**Authors:** Barbara Saavedra Deus, Gladstone Eustaquio de Lima Faria

**Affiliations:** ^1^ Private Clinic São Paulo Brazil

**Keywords:** botulinum toxin, eyebrow repositioning, facial aesthetics, incobotulinumtoxinA, orbicularis oculi

## Abstract

**Background:**

Eyebrow position plays a critical role in facial aesthetics and aging. Botulinum toxin is widely used for facial modulation, yet the role of treating completely the superior portion of the orbicularis oculi muscle in eyebrow repositioning remains underexplored.

**Objective:**

To evaluate the benefits, safety, and functional outcomes of botulinum toxin treatment in the superior portion of the orbicularis oculi muscle for eyebrow repositioning.

**Methods:**

Ten female participants with complaints of eyebrow dystopia were enrolled. Exclusion criteria included botulinum toxin treatment within the previous year. A standardized injection protocol with IncobotulinumtoxinA was applied, totaling 11 units per side. Patients were assessed with two‐dimensional photographs before and 15 days after treatment, GAIS scores, pain scale, and self‐administered questionnaires regarding adverse events and satisfaction.

**Results:**

Nine participants completed the study (mean age 32–61 years). At day 15, 89% reported improvement in eyebrow positioning (33.3% very much improved, 22.2% much improved, 33.3% improved), while 11.1% reported no change and none reported worsening. Mild pain was described by 77.8%, and 22.2% reported no pain. No significant adverse events, including eyelid ptosis or impaired lymphatic drainage, were observed. Overall satisfaction was 100%, and all participants indicated they would recommend the treatment.

**Conclusion:**

Isolated but broader treatment of the superior orbicularis oculi muscle achieved satisfactory eyebrow elevation without increasing adverse events. This technique may serve as a promising adjunct to established botulinum toxin protocols for improved eyebrow positioning.

## 1 Introduction

The position of the eyebrow is a highly relevant attribute in facial aesthetics and plays a significant role in gender differentiation. Female eyebrows are typically arched and located above the orbital rim, whereas male eyebrows tend to be straighter and positioned directly on the orbital rim [1]. During the aging process, regardless of sex, eyebrows tend to shift caudally, contributing to the loss of a youthful appearance, predisposing to pseudo‐ptosis of the eyelid, and accentuating dermatochalasis [2, 3, 4].

Botulinum toxin is widely used in the modulation of facial mimicry, not only attenuating expression lines but also providing control between elevator and depressor muscles, inducing selective muscle atrophy, and modifying facial vectorization [5].

In eyebrow positioning, the balance of muscular forces occurs between the frontalis muscle—the only elevator—and all the depressors, namely the procerus, corrugator supercilii, and orbicularis oculi [6, 7, 8]. The orbicularis oculi, a sphincter muscle, is a strong depressor of the eyebrow medially and laterally. While lateral canthus blockade is well established in the literature, superior blockade of the orbicularis may provide additional potential for eyebrow elevation [4, 9].

However, due to the potential risk of adverse effects such as eyelid ptosis, the use of botulinum toxin in the superior portion of the orbicularis oculi muscle raises concern among injectors. Therefore, the use of high‐precision botulinum toxin, in addition to injector expertise, is essential.

## 2 Objective

This study aimed to evaluate the benefits of treating the superior portion of the orbicularis oculi muscle for eyebrow repositioning, as well as to assess the aesthetic and functional improvements in ocular aperture.

## 3 Methods

Ten female participants presenting with eyebrow dystopia were selected. Written informed consent was obtained and all the ethical principles were taken. Exclusion criteria included botulinum toxin treatment within the previous year.

**FIGURE 1:**
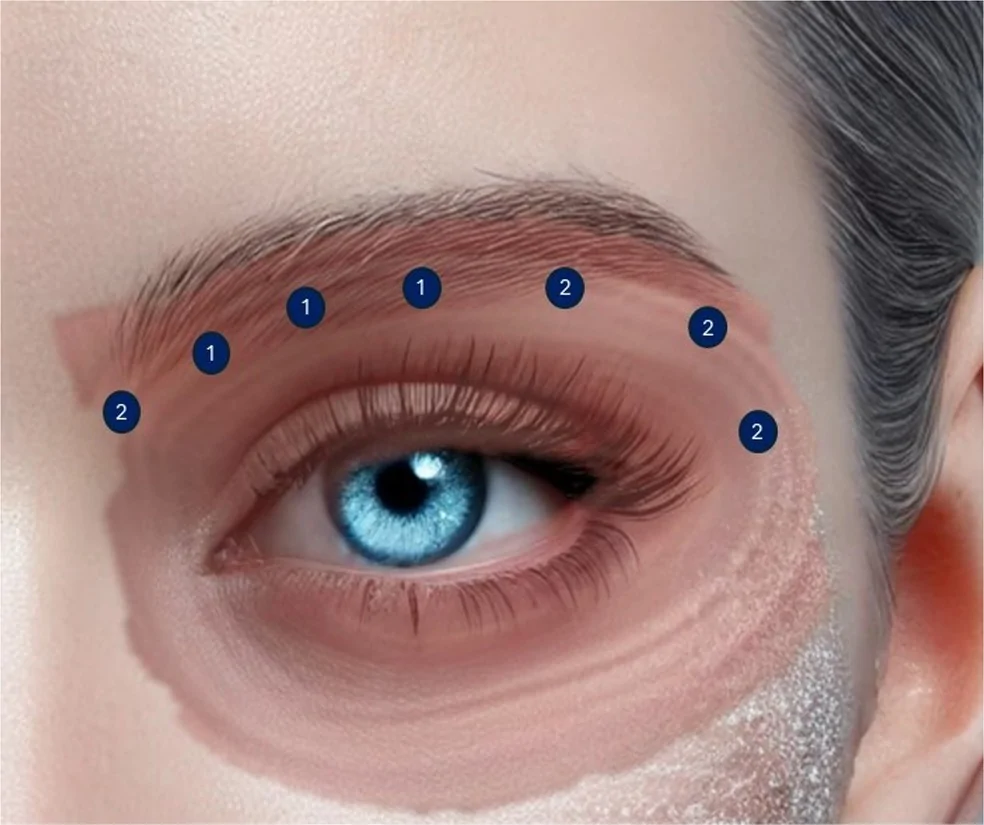
Injection technique for complete superior orbicularis oculi blockade. Seven points were marked from the superomedial to the lateral portion projected at the outer canthus. The first point was placed at the mid‐pupillary line, with three additional points medially and three laterally. The most medial point and the three lateral points each received 2 units of botulinum toxin, while the three points between the mid‐pupillary line and medial canthus received 1 unit each, totaling 11 units per side.

All participants were treated using the same injection scheme. Seven points were marked from the superomedial to the lateral portion projected at the outer canthus. The first point was placed at the mid‐pupillary line, with three additional points marked medially and three laterally. The most medial point and the three lateral points each received 2 units of IncobotulinumtoxinA, while the three points between the mid‐pupillary line and medial canthus received 1 unit each. In total, 11 units were applied per side. Injections were intramuscular, with emphasis on a nearly subdermal placement due to the intimate contact between skin and orbicularis oculi (Figure 1).

For treatment safety, IncobotulinumtoxinA (Xeomin, Merz Pharmaceuticals GmbH, Frankfurt, Germany) was used, as studies show it provides a smaller diffusion halo, greater precision, and reduced risk of spread to non‐target muscles [10], particularly the levator palpebrae superioris.

A 100‐unit vial of IncobotulinumtoxinA was reconstituted with 1 mL of 0.9% saline and administered using a 0.3 mL syringe (Becton, Dickinson and Company, Franklin Lakes, NJ, USA).

Participants were evaluated with two‐dimensional photographs before and 15 days after treatment. As an exploratory objective assessment, standardized two‐dimensional photographs were analyzed using a pixel‐based method to estimate changes in ocular aperture. The visible palpebral fissure area was compared before and after treatment by quantifying the corresponding pixel area in standardized images. This method was introduced as a pilot exploratory approach and was not intended to represent a validated measurement tool. Subjective evaluation was performed using the GAIS scale. A pain scale was applied immediately post‐treatment. On Day 15, the need for dose complementation was assessed. A questionnaire was sent via Google Forms to record adverse events and overall satisfaction.

All ethical principles of the Declaration of Helsinki were followed.

## 4 Results

Nine participants (mean age 32–61 years) completed the study; one was lost to follow‐up due to relocation.

**FIGURE 2:**
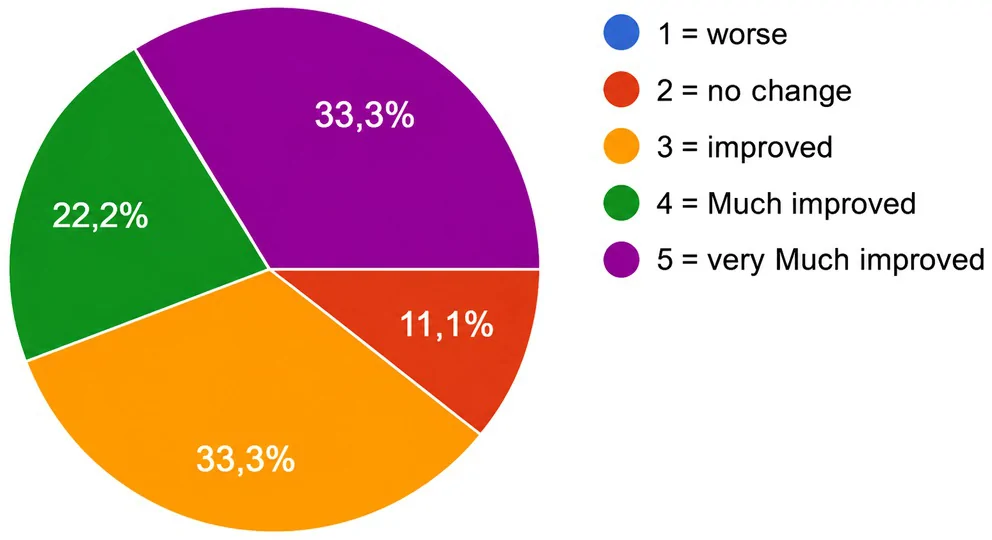
Global Aesthetic Improvement Scale among participants.

Regarding GAIS assessment, 33.3% reported very much improved, 22.2% much improved, 33.3% improved, 11.1% no change, and 0% worse (Figure 2).

For pain assessment, 77.8% reported mild pain and 22.2% reported no pain. No participants reported moderate, severe, or unbearable pain.

No significant adverse events were observed, such as eyelid ptosis, lagophthalmos, or lymphatic edema. Only one case of bruising and two cases of tenderness at the injection site were reported. No dose complementation was required.

High levels of patient satisfaction were observed, with all participants stating they would recommend the treatment.

Although GAIS and patient satisfaction scores demonstrated high rates of perceived improvement, these findings should be interpreted with caution, as subjective assessments are inherently susceptible to expectation and perception bias. Nevertheless, GAIS remains a widely accepted outcome measure in aesthetic studies and provides valuable insight into patient‐perceived aesthetic benefit. In the present study, subjective evaluations were complemented by exploratory objective assessment methods, including standardized photographic analysis and preliminary pixel‐based ocular aperture evaluation. Future studies with larger cohorts and validated objective measurement tools are necessary to further substantiate the reproducibility and magnitude of the observed effects.

**FIGURE 3:**
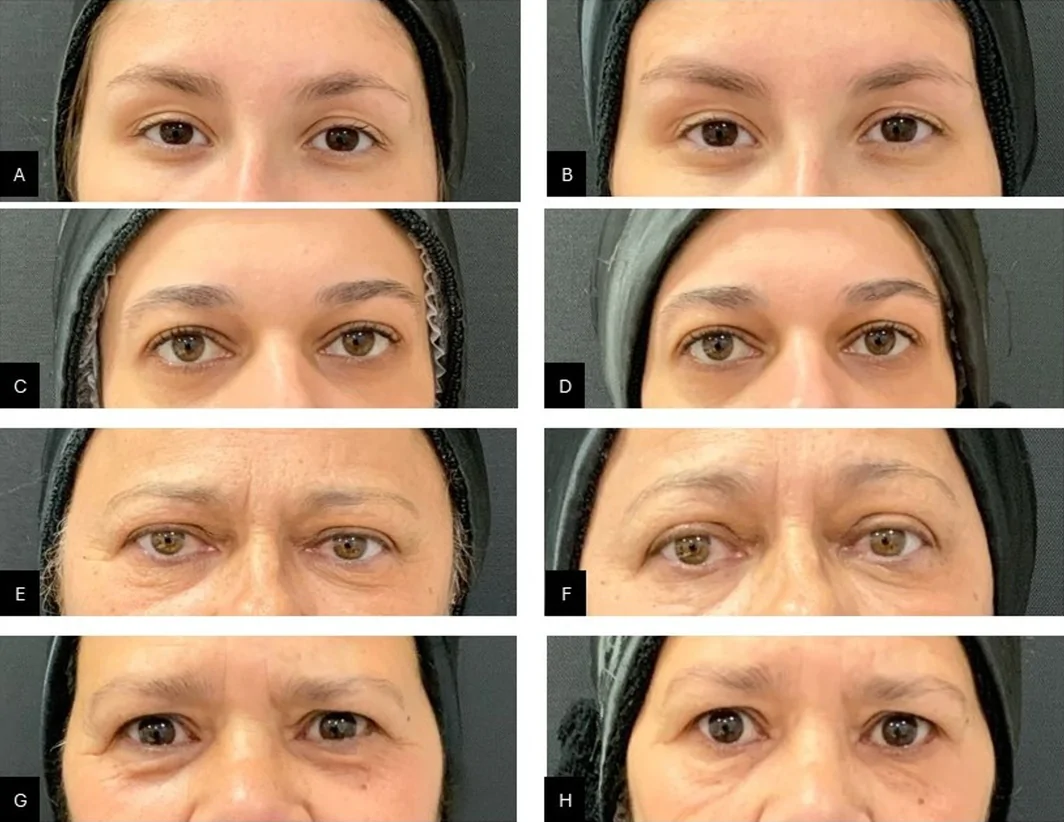
Before (A, C, E, G) and after (B, D, F, H) pictures of different participants with different ages and eyelid dystopia.

Pre‐ and post‐treatment results of study participants are presented, encompassing different age groups and degrees of eyebrow ptosis in Figure 3.

**FIGURE 4:**
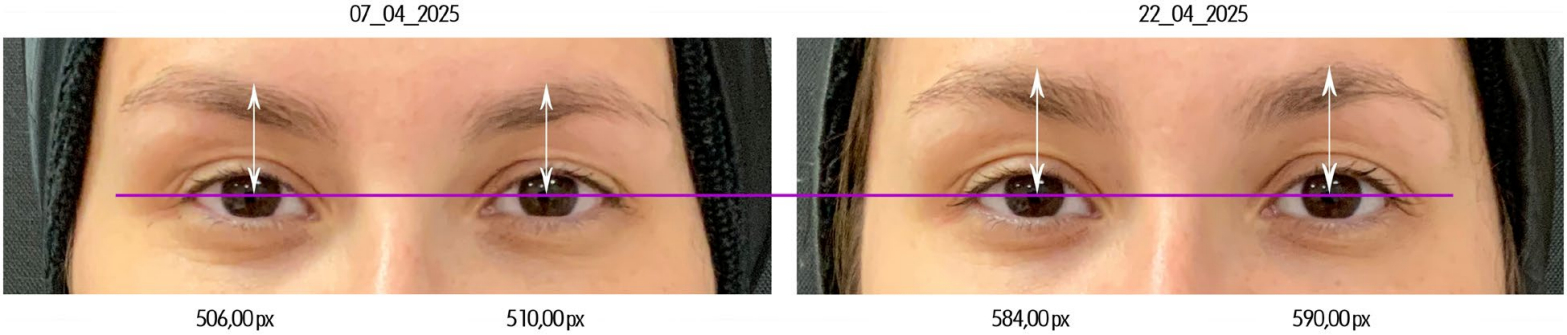
Pilot technique for eyelid dystopia assessment with pixel‐based increments.

A pilot evaluation is shown, employing pixel count gain as an objective method to assess the improvement achieved in Figure 4.

## 5 Discussion

The eyebrow plays a critical role in facial expression and beauty perception [4]. Ptosis of the eyebrow due to aging is common and is attributed to factors such as bone remodeling, fat pad changes, ligament laxity, and skin flaccidity [6].

Surgical correction can restore a youthful look but carries inherent risks, higher bruising incidence, and longer recovery compared with minimally invasive techniques. Thus, conservative approaches are increasingly sought [4].

The eyebrow is a dynamic structure, whose position is primarily determined by the balance of the upper facial musculature. The frontalis muscle is the main elevator of the brow [6, 7]. For this reason, in patients with brow ptosis and/or blepharochalasis, lower doses of botulinum toxin should be applied to the frontalis muscle in order to avoid further brow descent.

In contrast, the corrugators, procerus, depressor supercilii, and the medial fibers of the orbicularis oculi act to lower the medial brow. Therefore, botulinum toxin application in these muscles counteracts this movement and enables medial opening of the eyes. This was demonstrated in the study by Carruthers and Carruthers [11], in which treatment was limited to the glabellar complex, resulting in a significant impact on the medial brow position.

The most common approach to treating the orbicularis oculi muscle is targeting its lateral portion. Being a sphincteric muscle, its contraction occurs through a constrictive mechanism, leading to lateral brow descent [12]. Adequate relaxation of this portion allows elevation of the brow tail, a benefit clearly demonstrated in the study by Ahn et al. [13], in which treatment was performed exclusively on the lateral orbicularis oculi.

The combined treatment of the glabellar complex and the lateral orbicularis oculi provides an enhanced repositioning of the eyebrow, as it promotes both medial and lateral opening [8, 14].

An imperative question at this point is whether relaxation of the entire superior portion of the orbicularis oculi muscle could also contribute to brow elevation, and whether the lack of standardized techniques, along with concerns about severe complications such as eyelid ptosis, has limited the clinical use of this approach.

In clinical practice, it is uncommon to treat only a single muscle group, even when this corresponds to the patient's primary complaint. Rather, treatment strategies are typically designed to balance the activity of the elevator (frontalis) and depressor muscles (procerus, corrugator, and orbicularis oculi), thereby achieving a vectorization of muscular forces in an antigravitational direction and, consequently, a brow lift.

In this study, we explored whether broader treatment of the superior orbicularis oculi could improve eyebrow positioning without added complications. Indeed, 89% of participants observed improvement, with no reports of ptosis or impaired drainage.

The choice of IncobotulinumtoxinA was important, given its reduced diffusion halo and precision [10].

Limitations of this study include the small sample size, the short follow‐up period, and the challenge of objectively quantifying eyebrow repositioning and ocular aperture changes. An exploratory pixel‐based analysis was used as a preliminary objective assessment method to estimate changes in ocular aperture. Although promising as a reproducible and accessible tool, this technique has not yet been validated and should therefore be interpreted as a pilot methodological proposal requiring further standardization and validation in larger studies.

Another limitation of this study is the absence of a control group. Although a split‐face design using the contralateral side as an internal control could theoretically provide a comparative assessment, this approach was considered inappropriate in the present study due to ethical and aesthetic concerns. Deliberately inducing temporary eyebrow asymmetry could result in noticeable facial imbalance and potential patient distress, which may not be ethically acceptable in aesthetic interventions. Therefore, all participants were treated bilaterally in order to preserve facial harmony and ensure a clinically acceptable outcome. Consequently, this study was designed as a pilot proof‐of‐concept investigation focused primarily on safety, feasibility, and preliminary efficacy.

Future studies with larger cohorts and combined techniques are encouraged.

## 6 Conclusion

Broader treatment of the superior orbicularis oculi muscle achieved satisfactory eyebrow elevation without increasing adverse events. This approach may represent a promising adjunct to established botulinum toxin protocols for eyebrow repositioning.

## Author Contributions

B.S.D., G.E.d.L.F. performed and designed the research, data analysis, and wrote the article.

## Funding

The authors have nothing to report.

## Ethics Statement

All procedures performed in studies involving human participants were conducted in accordance with the ethical standards of the institutional and/or national research committee and with the 1964 Declaration of Helsinki and its later amendments or comparable ethical standards.

## Consent

Informed consent was obtained from all individual participants included in the study.

## Conflicts of Interest

The authors declare no conflicts of interest.

## Data Availability

Data sharing not applicable to this article as no datasets were generated or analysed during the current study.
